# Molecular Mechanisms Underlying β-Adrenergic Receptor-Mediated Cross-Talk between Sympathetic Neurons and Immune Cells

**DOI:** 10.3390/ijms16035635

**Published:** 2015-03-11

**Authors:** Dianne Lorton, Denise L. Bellinger

**Affiliations:** 1 College of Arts and Sciences, Kent State University, Kent, OH 44304, USA; 2 Department of Human Anatomy and Pathology, Loma Linda University, School of Medicine, Loma Linda, CA 92350, USA; E-Mail: dbellinger@kent.edu

**Keywords:** Neural–immune interactions, stress, β_2_-adrenergic receptor signaling, GRK, β-arrestin, PKA, ERK1/2, receptor regulation, innate and adaptive immunity

## Abstract

Cross-talk between the sympathetic nervous system (SNS) and immune system is vital for health and well-being. Infection, tissue injury and inflammation raise firing rates of sympathetic nerves, increasing their release of norepinephrine (NE) in lymphoid organs and tissues. NE stimulation of β_2_-adrenergic receptors (ARs) in immune cells activates the cAMP-protein kinase A (PKA) intracellular signaling pathway, a pathway that interfaces with other signaling pathways that regulate proliferation, differentiation, maturation and effector functions in immune cells. Immune–SNS cross-talk is required to maintain homeostasis under normal conditions, to develop an immune response of appropriate magnitude after injury or immune challenge, and subsequently restore homeostasis. Typically, β_2_-AR-induced cAMP is immunosuppressive. However, many studies report actions of β_2_-AR stimulation in immune cells that are inconsistent with typical cAMP–PKA signal transduction. Research during the last decade in non-immune organs, has unveiled novel alternative signaling mechanisms induced by β_2_-AR activation, such as a signaling switch from cAMP–PKA to mitogen-activated protein kinase (MAPK) pathways. If alternative signaling occurs in immune cells, it may explain inconsistent findings of sympathetic regulation of immune function. Here, we review β_2_-AR signaling, assess the available evidence for alternative signaling in immune cells, and provide insight into the circumstances necessary for “signal switching” in immune cells.

## 1. Introduction

The sympathetic nervous system (SNS) communicates with all cells of the immune system and supporting stromal cells. Communication with immune cells occurs directly by neurotransmitter release from sympathetic nerves that bind to postsynaptic receptors expressed on immune cells. Indirect effects of sympathetic activity may occur by regulating cytokine release or stromal cell function. The purpose of SNS–immune communication is to maintain immune homeostasis under basal conditions, augment host defense to eliminate pathogens, promote healing after tissue injury, and restore homeostasis after pathogen elimination or tissue repair. The SNS-immune pathway also provides “hardwired” circuitry through which psychological and social factors can profoundly adapt host immunity and healing. Sympathetic–immune regulation is mediated largely by stimulated release of its major neurotransmitter, norepinephrine (NE) and subsequent intercellular signaling via postsynaptic adrenergic receptors (ARs) expressed in closely apposed immunocytes (*i.e*., T and B lymphocytes, antigen-presenting cells, stromal cells, granulocytes, macrophages, and mast cells).

Under normal physiological conditions, SNS–immune regulation is adaptive, influencing the normal immune response to injury or foreign antigens/toxins/infectious agents, with subsequent restoration of homeostasis and recovery to a healthy state [1,2]. Importantly, resolution and return to homeostasis requires that the SNS can up-regulate and down-regulate cellular expansion, differentiation and effector cell functions at appropriate times during the immune response. In order for the SNS to appropriately regulate the immune system across all of these settings, it must be highly adaptive in two respects. First the signaling pathways it uses must be able to up- and down-regulate diverse target cell functions across time (*i.e*., expansion, differentiation, apoptosis, and cytokine secretion), and secondly, its signaling pathways must be able to intersect with the diverse signaling pathways that mediate these cellular functions. This notion is consistent with a confusing literature documenting that the SNS can increase or decrease most immune measures. Recent findings begin to unravel the cellular mechanisms that may explain this duality in function under normal and pathologic conditions. Moreover, prolonged or inappropriate activation of either the SNS or immune system can result in the failure of the immune and sympathetic nervous systems to shut-off immune responses and to re-establish immune system homeostasis within normal physiological ranges. Under such conditions the immune system and/or SNS can promote pathological and lethal effects, including chronic inflammation, toxic shock, tissue damage, immune deficiency, autoimmunity and cancer.

New information has emerged regarding mechanisms through which intercellular communication occurs between sympathetic nerves and its AR-expressing target cells that may explain the ability of the SNS to both inhibit and enhance immune responses depending upon the context of the immune response. Much of the new understanding of how these G protein-coupled receptors (GPCRs) activate both inhibitory and stimulatory signaling pathways has not yet been extended to understanding SNS regulation of functions in immune cells. Most research regarding sympathetic regulation of immune cell functions has focused on regulation via β_2_-ARs, thus, we will focus this review on this receptor subtype. Historically, β_2_-ARs were thought to exert largely inhibitory signals to cells of the immune system by inducing cAMP and protein kinase A (PKA). It is now clear that signaling via β_2_-ARs is much more complex and can, in addition to their traditional signaling pathway, activate multiple signal transduction pathways to exert inhibitory and/or enhancing effects on cell functions. These receptors, like other GPCRs, are now viewed as complex, multidimensional activators of a variety of potential signaling cascades rather than simply activating binary inhibitory or stimulatory signaling pathways via coupling to G proteins. In this paper, we review current information on β_2_-AR traditional (canonical) and nontraditional (non-canonical) signal transduction pathways, then discuss the possibility that nontraditional signal pathways identified for β_2_-ARs are involved in sympathetic regulation of immune cell functions.

Over the past decade, it has become clear that ARs and other GPCRs can activate non-traditional signal transduction pathways in addition to their traditional signaling pathways [3,4,5,6]. This non-traditional signaling occurs in a cell type-dependent and G protein-independent manner [3,4,5,6]. Most studies demonstrating activation of non-traditional signaling by ARs have been done using *in vitro* methods with various cell lines and have largely focused on β_2_-ARs in non-immune cells. The extent to which activation of non-canonical signaling pathways via β_2_-ARs is physiologic or pathologic or whether they are functional signaling pathways in immune cells is not clear. Here, we review the current information on the traditional and non-traditional mechanisms through which β_2_-ARs signal, how β_2_-AR functions are regulated by SNS nerve firing (SNS activity) and cross-talk with other signaling pathways activated by immune challenge, and the existing evidence for non-canonical signaling via β_2_-AR in immune cells. Recent findings from our group and others do support that a “switch” in signal from canonical to non-canonical pathways for β_2_-AR can occur in immune cells under inflammatory conditions. Further, the functional implications for signaling via non-canonical pathways with regard to immune functions and the clinical relevance of understanding how β_2_-ARs are regulated are discussed.

## 2. Canonical Intracellular Signaling by β_2_-Adrenergic Receptors (ARs)

### 2.1. cAMP: The Second Messenger in the β_2_-AR Signaling Pathway

**Figure 1:**
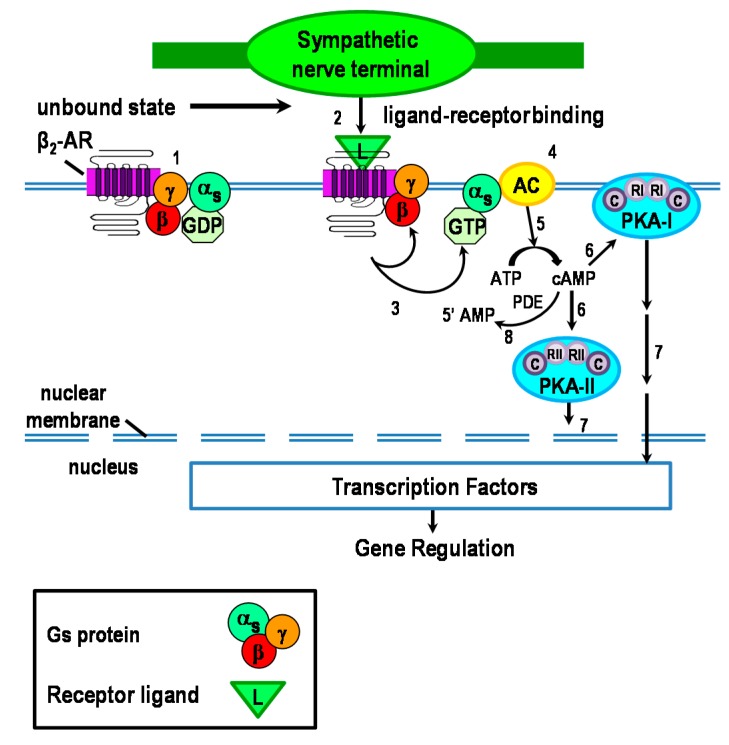
The canonical β_2_-adrenergic receptor (AR) signaling pathway is illustrated here. In target tissues, activated sympathetic nerves release the neurotransmitter, norepinephrine (NE) from “boutons en passage”. NE and epinephrine from the circulation are the natural ligands (L) for the β_2_-AR. β_2_-ARs couple with stimulatory Gα_s_ subunit to modulate the activity of adenylate cyclase (AC). Receptor activation causes dissociation of Gα_s_ from the Gβγ subunit complex of the Gs protein, which results in AC-mediated production of cAMP from ATP. Next, cAMP activates either protein kinase A-I (PKA-I) or -II. PKA activation leads to the activation of transcription factors such as cAMP response element-binding (CREB) protein to regulate gene transcription.

In the canonical pathway, β_2_-ARs are coupled to Gα_s_, which activates cAMP-PKA-mediated intracellular signaling (Figure 1) [7,8,9,10]. Briefly, NE binds to β_2_-ARs expressed in immune cells (Figure 1, #1–2). Ligand binding induces the guanosine diphosphate–guanosine triphosphate (GDP–GTP) exchange, and Gα_s_ and Gβγ (G protein alpha (s subtype) and beta/gamma subunits, respectively) dissociation from each other (Figure 1, #3). GTP-Gα_s_ is recruited to the membrane-associated lipid raft, and subsequently activates adenylate cyclase (AC) present in distinct subdomains of the plasma membrane and cytoplasm (Figure 1, #4). AC catalyzes the conversion of ATP to cAMP (Figure 1, #5) [11], the second messenger for β_2_-ARs [12,13]. Cyclic-AMP activates and regulates PKA, of which there are two isoforms that differentially localize to either the cell membrane (PKA-I) or intracellularly (PKA-II) (Figure 1, #6; discussed further below). PKA mediates most of the resulting gene transcription (Figure 1, #7). However, cAMP can activate gated ion channels, and exchange proteins activated by cAMP (exchange protein directly activated by cAMP (Epac); not shown) [14]. Signal transduction is terminated by degradation of cAMP by phosphodiesterases (PDE) (Figure 1, #8).

There are nine different membrane-bound isoforms and one soluble isoform of class III AC, all of which are activated by Gs. Each isoform is differentially expressed depending on the specific type of cell. For example, immune cells express high amounts of the AC7 isoform and low amounts of AC3, 6 and 9 [15]. Each AC isoform alters cell function in a specific manner, in part due to where they reside in the cell. For instance, Ca^2+^-insensitive AC7 is excluded from lipid rafts [16], but not Ca^2+^-sensitive AC3 and AC6 [7,8,9]. This leads to location-restricted pools of cAMP that can selectively target molecules to mediate distinct physiological outcomes. This may explain, in part, the many varied responses of the large number of GPCRs that are coupled to cAMP. Interestingly, current evidence indicates the β_2_-AR resides outside lipid rafts, while Gs and AC can reside either within or outside lipid rafts. At present the functional link between the receptor and the segregation of its signaling molecules in regulation of β_2_-AR function is poorly understood. However, existing data indicate that segregation of these signaling molecules within lipid rafts restrain β_2_-AR activity and function to regulate receptor responsiveness [17]. AC7, an AC excluded from lipid rafts, is the major isoform that regulates cAMP synthesis in macrophages and T and B lymphocytes [18]. Studies using mice deficient in AC7 indicate that this isoform is required for optimal macrophage and T and B lymphocyte functions during innate and adaptive immunity [15].

### 2.2. Intracellular Protein Kinase A (PKA) Localization Determines Specificity of Response

**Figure 2:**
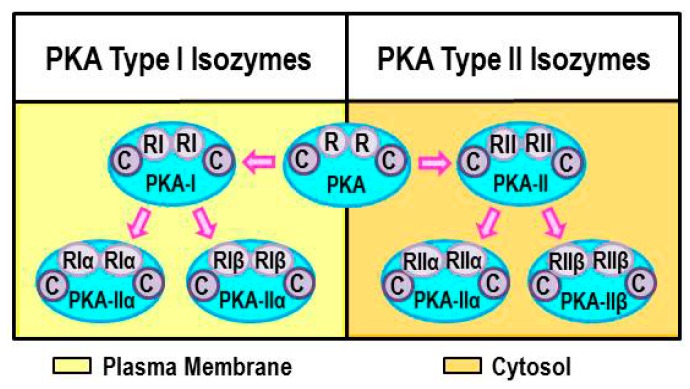
Illustration of the structure and current nomenclature for protein kinase A (PKA) Isoforms. Isoforms of PKA differ in the regulatory proteins (R) they express, either regulator protein I (RI) or II (RII) for PKA-I or II isoforms. PKA-I associates with the plasma membrane, whereas PKA-II localizes to cytosol and the membranes of cell organelles. Proteins called A-kinase anchor proteins (AKAPs) that bind PKAs are responsible for the site-specific localization of PKA isoforms.

PKA is composed of a regulatory subunit dimer (R), which is bound to a catalytic subunit (C) (Figure 2). Two major types of regulatory subunits occur in mammals, designated PKA RI and RII (four isoforms, RIα, RIβ, RIIα, and RIIβ, have been identified). The regulatory subunits bind cAMP, an event that releases the catalytic subunits. The catalytic subunits, once released from the regulatory subunits, catalyze the transfer of ATP terminal phosphates to serine, or threonine residues in target proteins. Phosphorylation of the targeted protein changes its functional state (resting/activated). The regulatory subunit is also critical for localizing PKA within specific intracellular compartments; PKA-RI and PKA-RII localizes to the plasma or organelle membranes and cytosol, respectively (Figure 2; PKA-I left panel, PKA-II right panel). Localization of PKA to specific compartments within the cell allows for the specific targeting of PKA substrates to be regulated by PKA-mediated phosphorylation. Specifically, the regulatory subunits of PKA bind to a structurally diverse group of A-kinase anchor proteins (AKAPs) that direct the intracellular location of PKA (e.g., plasma membrane, cytosolic or nuclear compartments.) [19,20,21]. Lymphocytes express all four PKA isoforms, however, the RIα types are the predominant isotypes expressed in T cells and in the spleen and thymus [22]. The RIα PKA isoform is required for normal immune functions [22,23,24]. In contrast, immune functions are normal in RIIα-knockout mice [22]. AKAPs also tether AC to PKA, gated-ion channels, or Epac. Most β_2_-AR-mediated effects in immune cells are attributed to activation of PKA. Much less is known regarding AR signaling through cAMP-induced Epac regulation of immune cells. This topic was recently reviewed by Shirshev [14] and will not be covered in this review.

### 2.3. Regulation of Canonical β_2_-AR Signaling

**Figure 3:**
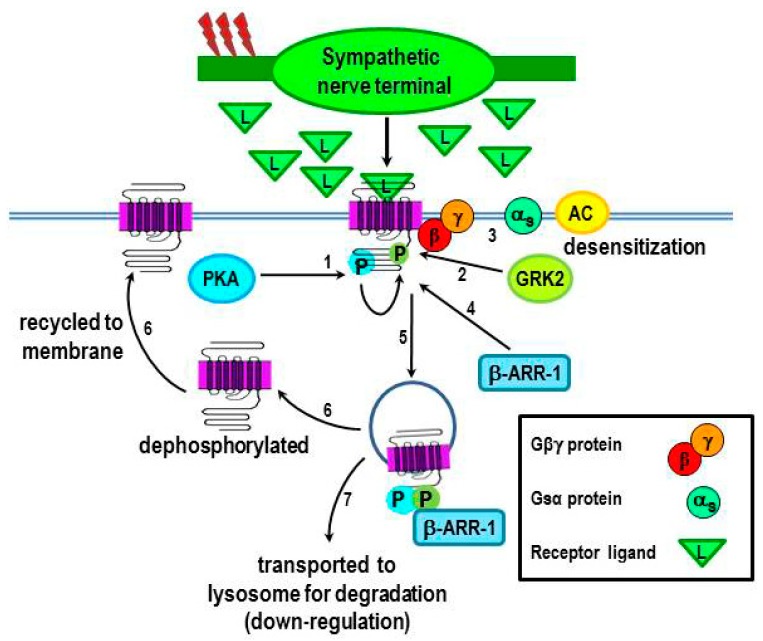
Receptor responsiveness to β_2_-AR ligands is regulated by protein kinases that phosphorylate the receptor. PKA that is activated after β_2_-AR activation by ligand (L) subsequently phosphorylates the receptor (#1), which uncouples Gα_s_ from the Gβγ subunit, this results in an uncoupling of Gα_s_ from the receptor (#3), thus terminating receptor signaling. Phosphorylation of the receptor by PKA also facilitates receptor phosphorylation by GRK2 (#2), which further drives receptor desensitization (#3). GRK2 recruits β-arrestin-1 to the receptor (#4), a step that leads to receptor internalization (#5). The internalized β_2_-AR is then transported to lysosomes for degradation (#7), receptor down-regulation or dephosphorylation and recycled to the cell membrane (#5–6). Chronic stimulation of the receptor or high ligand concentrations promote the transport of the receptor to lysosomes for degradation, resulting in receptor down-regulation. Red lightning bolts indicate increased sympathetic nerve firing.

Under inflammatory conditions, proinflammatory cytokines act in the hypothalamus to increase SNS tone in relevant tissues/organs. Increased NE release from sympathetic nerves in target tissue, and subsequent spillover into the circulation coordinates the localized and systemic immune responses, respectively [1]. Neuroendocrine release of epinephrine from the sympathoadrenal axis contributes to the systemic response. Increases or decreases in local NE concentrations dynamically down- or up-regulate β_2_-AR expression, respectively, which affects receptor function. Chronic stimulation or high NE concentrations activate the β_2_-AR and induce receptor desensitization and down-regulation in target cells, including cells of the immune system. Receptor desensitization and down-regulation result from β_2_-AR phosphorylation by PKA and transiently by G protein-coupled kinases (GRKs), specifically GRK2. PKA and GRK2-mediated phosphorylation target different serine sites on the carboxy tail of the β_2_-AR [25,26,27,28,29,30]. Enzyme-mediated phosphorylation determines the receptor responsiveness, as well as, their numbers expressed on the cell surface [31]. An overview of this process is shown in Figure 3. PKA-receptor phosphorylation (Figure 3, #1), which dissociates Gβγ from Gα_s_ (Figure 3, #2) facilitates GRK2-receptor phosphorylation at a different serine site by allowing GRK2 to bind with the Gβγ subunit (Figure 3, #3). Thus, desensitization involves a multi-step process, in which Gs signaling is “turned off”, and Gi transiently couples to the receptor via the Gβγ subunit (not shown; discussed below). Recruitment of the scaffolding protein, β-arrestin-1 to the receptor (Figure 3, #4) then induces receptor internalization (Figure 3, #5). Internalized receptors are either dephosphorylated and returned to the membrane (Figure 3, #6) or transported to lysosomes for degradation. These kinase-mediated actions on the β_2_-AR are the basic mechanisms regulating receptor function, expression, and localization in all cells that have been examined, including immune cells, and are essential for homeostatic regulation. This process is discussed in greater detail in the following subsection.

#### 2.3.1. β_2_-AR Desensitization

GRKs play a crucial role in agonist-induced desensitization of the β_2_-AR after canonical signaling. Seven GRKs (GRK1–7) comprise the currently known subtypes of this family of kinases [32]. GRK2 phosphorylates specific serines of the β_2_-AR, which creates a binding site for β-arrestin [29,33,34,35]. Phosphorylation of the β_2_-AR by GRK2 changes the conformation of the receptor, which allows β-arrestin to bind to the carboxy-terminal tail of the receptor [30,36,37]. The β-arrestin binding further alters the conformation of the β_2_-AR, which impedes Gs protein coupling to the receptor, and prevents receptor signaling via cAMP. This process is referred to as β_2_-AR desensitization [30].

#### 2.3.2. β_2_-AR internalization and Functional Consequences

As a consequence of β_2_-AR phosphorylation and subsequent β-arrestin binding, the agonist-bound β_2_-ARs are internalized [38,39] (Figure 3). Internalized β_2_-ARs undergo one of two fates. They may be dephosphorylated and recycled to the plasma membrane (Figure 3, #6), or degraded (Figure 3, #7), the latter being responsible for receptor down-regulation [40]. Receptor desensitization, degradation and recycling are important for maintaining homeostasis and ensuring fine tuning of the response being modulated. Malfunction of the β_2_-AR desensitization process has been linked to various diseases, including heart failure [41,42,43], asthma [44], and autoimmune diseases [45,46].

#### 2.3.3. PKA Phosphorylation Induces a β_2_-AR Switch in Coupling from Gs to Gi Protein

**Figure 4:**
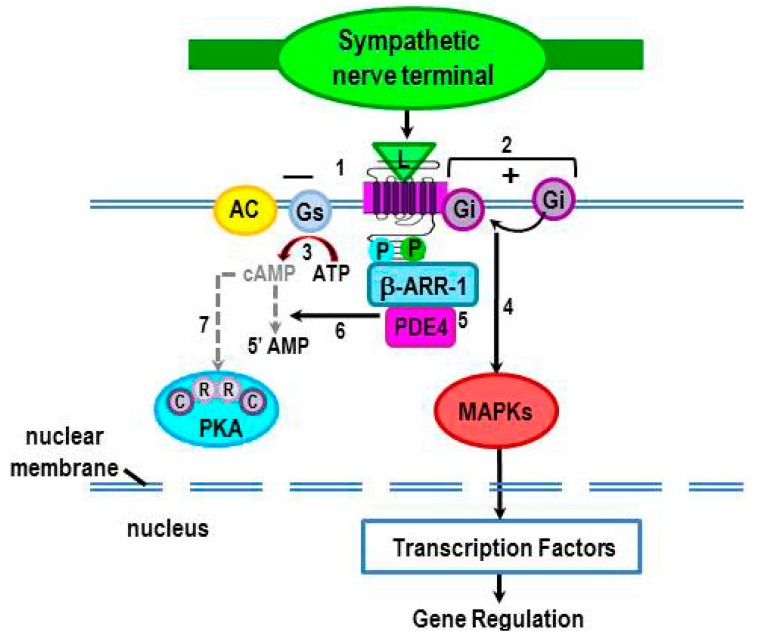
Phosphorylation of β_2_-ARs by PKA induces a conformational change that impairs Gs binding (#1), but enhances Gi binding (#2) to the receptor. This transient shift in receptor coupling prevents activation of AC and thus, inhibits signaling via cAMP (#3). Coupling of Gi to the receptor results in activation of mitogen-activated protein kinase (MAPKs) (#4). MAPKs subsequently phosphorylate transcription factors that regulate gene expression, including the activation of critical genes that drive innate and adaptive immunity. Binding of β-arrestin to the PKA and G-protein coupled receptor kinase 2 (GRK2)-phosphorylated receptor recruits phosphodiesterase (PDE) 4 to the receptor (#5). PDE4 degrades cAMP (#6), resulting in a reduction of local levels of cAMP, thus terminating cAMP activation of PKA (#7).

As indicated above, β_2_-AR phosphorylation by PKA (pβ_2_-AR_PKA_) alters the receptor conformation, which impairs Gs binding to the β_2_-AR (Figure 4, #1), but enhances β_2_-AR coupling to Gi protein (Figure 4, #2). Both of these events prevent the production of cAMP (Figure 4, #3). In human embryonic kidney (HEK)-293 cells, Gi coupling to the receptor (Figure 4, #2) induces transient signaling through mitogen-activated protein kinase MAPK pathways (Figure 4, #4) [47,48]. Whether transient activation of Gi-mediated MAPK pathways occurs in cells of the immune is not known. Three MAPK subgroups—extracellular signal-regulated protein kinase (ERK), c-Jun *N*-terminal kinase (JNK), and p38 MAPK—phosphorylate many transcription factors and other kinases that are important regulators for gene expression [49,50,51,52], including the activation of critical genes that drive innate [53,54,55] and adaptive immunity [55,56,57,58,59].

#### 2.3.4. Terminating cAMP Signal Transduction

Binding of β-arrestin to the β_2_-AR recruits cyclic nucleotide PDE to the receptor-β-arrestin complex (Figure 4, #5) [60,61,62,63,64]*.* PDEs are a family of enzymes that hydrolyze cAMP to nucleotide 5'-monophosphate (5'-AMP) by degrading the phosphodiester bond (Figure 4, #6). Thus, when PDE is recruited to the receptor, it reduces local levels of cAMP and terminates second messenger signaling from the previous β_2_-AR stimulation. Hydrolyzing cAMP “shuts off” its ability to transduce downstream signaling (Figure 4, #7). Thus, PDE regulates intracellular cAMP (cAMP_i_) levels, and therefore, the amplitude and duration of the second messenger response. PDE also establishes gradients within localized subcellular compartments or domains particularly in compartments where PDE concentrations are high [18].

There are eleven families of PDEs, of which three specifically hydrolase cAMP–PDE4, 7 and 8 [65,66]. PDEs 5, 6 and 9 selectively hydrolyze cGMP. PDEs 1–3, 10 and 11 degrade both cAMP and cGMP, providing a means for cross-regulation between pathways that use these second messengers. Selective inhibitors of phosphodiesterases have been identified that target cAMP and/or cGMP. These inhibitors prevent cyclic nucleotide breakdown, and therefore prolong the activity of cAMP and/or cGMP. Selective PDE inhibitors of cAMP mimic sympathetic activation. This is an indirect route in which PDE inhibitors could be used to regulate immune function. PDE4 is the major PDE family member expressed in inflammatory and immune cells, providing a second, more direct target for selective PDE4 inhibitors to drive anti-inflammatory processes [60]. Currently, these inhibitors are used in the treatment of chronic inflammatory disorders, in addition to their use as research tools to block the breakdown of cyclic nucleotides in immune cells after stimulation with GPCRs *in vitro* [67]*.*

The regulation of β_2_-ARs by GRKs and β-arrestins plays essential roles in maintaining the normal functions of cells and tissues, including the immune cells. Cells of the immune system express high levels of GRK2, exceeding levels in the heart by 4–5-fold [68]. In addition to GRK2, immune cells also express GRKs 3, 5, and 6 [68,69,70], but these GRKs are less well studied than GRK2. Down-regulation and desensitization of the β_2_-AR is observed in many diseases where net SNS firing rate (activity) is chronically elevated and robust/chronic inflammation ensues, including hypertension [71], sepsis [72,73,74], rheumatoid arthritis (RA) [75,76,77], and asthma [78,79,80]. For hypertension, changes in lymphocyte β_2_-AR density and poorer responsiveness are significant predictors of cardiovascular mortality and myocardial infarction [71]. In fact, treatment of rats with a β_2_-AR agonist for 2 weeks equally enhances expression of cardiac and lymphocyte expression of GRK2 mRNA in the heart and lymphocytes [81]. This report is consistent with a previous one demonstrating that an increase in GRK2 expression occurs in peripheral blood mononuclear cells (PBMCs) from hypertensive patients [82].

Reduced GRK2 levels are also observed in PBMCs from patients with RA [83] and multiple sclerosis (MS), as well as in animal models of these autoimmune diseases like adjuvant-induced arthritis (AA) [83] and experimental autoimmune encephalomyelitis (EAE) [84]. In contrast to RA, it is interesting that MS patients have a different signaling profile. In patients with relapsing-remitting MS, the β_2_-AR density and its capacity to produce cAMP in PBMCs are increased, not shut-off as in RA. These findings in MS patients strongly correlate with disease activity [85,86,87]. Mechanistically, these data support chronic β_2_-AR up-regulation, deficient GRK2 expression, and increased cAMP production as a consequence of PKA-mediated β_2_-AR phosphorylation in MS. Consistent with these findings, GRK2(+/−) mice, which express reduced GRK2, do not develop relapsing and remitting EAE that is observed in the wild-type EAE mice [84]. The absence of relapses in GRK2 (+/−) mice is associated with a marked reduction in inflammatory infiltrates in the central nervous system (CNS). These examples indicate the need for additional research to further our understanding of β_2_-ARs and other GPCR signaling, and their regulation by GRK2 in autoimmunity.

## 3. Non-Canonical Intracellular Signaling by β_2_-ARs: G Protein-Independent Signaling

While β_2_-AR internalization is well recognized to homeostatically attenuate receptor responsiveness, there is mounting evidence that the endocytic pathway can also generate receptor-initiated signals that are G protein-independent. These studies reveal that GRKs are involved in initiating a GPCR-mediated, β-arrestin-dependent, G protein-independent signaling pathway, including β_2_-ARs. This β-arrestin-dependent signaling pathway induces physiological responses that differ from the canonical G protein-mediated responses [88,89,90]. In non-canonical GPCR signaling, the carboxy-terminus of the β_2_-AR is serine/threonine-specifically phosphorylated by different GRK subtypes. Non-canonical signaling induces the activation of alternative signaling pathways that typically oppose canonical signaling by β_2_-ARs. Site-specific phosphorylation by specific subtypes of GRKs determines whether the GPCR undergoes desensitization or signals via an alternative pathway [91]. Nobles and coworkers [91] find that the subtype of GRK that phosphorylates the receptor determines the functional role of β-arrestin. Phosphorylation by GRK2 induces β-arrestin-mediated desensitization, whereas phosphorylation by GRK5 or 6 results in β-arrestin-mediated signaling. Phosphorylation of the β_2_-AR by GRK2 or 5/6 is dependent on the concentration of the agonist [27,92,93]. High agonist concentrations induce GRK-5/6- rather than GRK-2-mediated phosphorylation of the β_2_-AR.

Over the last decade, it has been repeatedly demonstrated in various cell types that β_2_-ARs can signal via G protein-independent, β-arrestin-mediated non-canonical signaling pathways. Four arrestin subtypes are identified, arrestins 1–4. Of these, β-arrestin 1 and 2 are present in all mammalian cells examined [94]. Both β-arrestin 1 and 2 can induce β_2_-ARs desensitization and internalization [95]. However, β-arrestin 1 is linked to cAMP-PKA signaling, while β-arrestin 2 couples GPCRs to MAPK pathways [96]. Signaling of β_2_-ARs through β-arrestin 2 induces sustained ERK1/2 rather than the traditional cAMP-PKA signaling [29,31,38,48,92].

**Figure 5:**
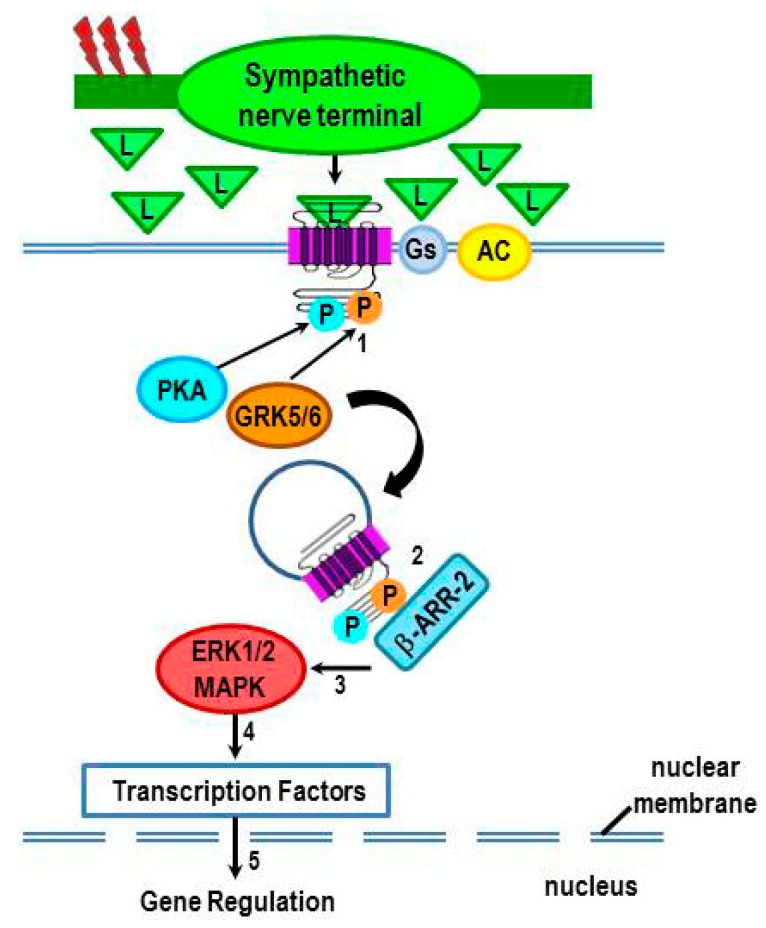
Phosphorylation of β_2_-AR by GRK-2, -5, and -6 occurs in an agonist concentration-dependent manner to induce different receptor functions. High sympathetic firing activity (red lightning bolts) drives the β_2_-AR-Gs-independent (non-canonical) pathway by flooding the extracellular space with ligand (L, *i.e.* NE). Chronic receptor activation induces not only PKA-mediated phosphorylation of the receptor, but also phosphorylation by GRK5/6 (#1) instead of GRK2. Receptor phosphorylation by PKA and GRK5/6 promote receptor desensitization and internalization by recruiting β-arrestin 2 (β-ARR-2) to the receptor (#2). Once bound to the receptor, β-arrestin-2 acts as a scaffold for the sustained activation of the MAPK, ERK 1/2 (#3). Beta-arrestin activation of ERK1/2 MAPK, in turn, increases the translocation of transcription factors (#4) into the nucleus to influence gene transcription (#5).

Similar to GRK-2-induced β-arrestin 1 binding to β_2_-ARs, β-arrestin 2 binds to the β_2_-AR after its phosphorylation by GRK 5/6 (Figure 5, #1), but in a manner that sets up a scaffolding for MAPK activation (Figure 5, #2–3) [48,91,96,97,98,99]. MAPK activation induces the activation of transcription factors, which travel to the nucleus to alter gene transcription (Figure 5, #4–5). In this manner, high agonist concentrations can induce sustained ERK activation independent of the G protein pathways [48,100,101]. Therefore, chronically elevated sympathetic tone may provide conditions for differential phosphorylation of β_2_-AR that result in sustained β_2_-AR-induced ERK1/2 signaling. Thus, signal transduction via ERK1/2 may explain why β_2_-AR stimulation can up- or down-regulate certain responses like lymphocyte proliferation or production of certain cytokines in an immune context-dependent manner, particularly during conditions of high SNS tone.

## 4. Immune System–SNS “Cross-Talk”

### 4.1. Cross-Talk and the Canonical Pathway: Traditional Viewpoint

**Figure 6:**
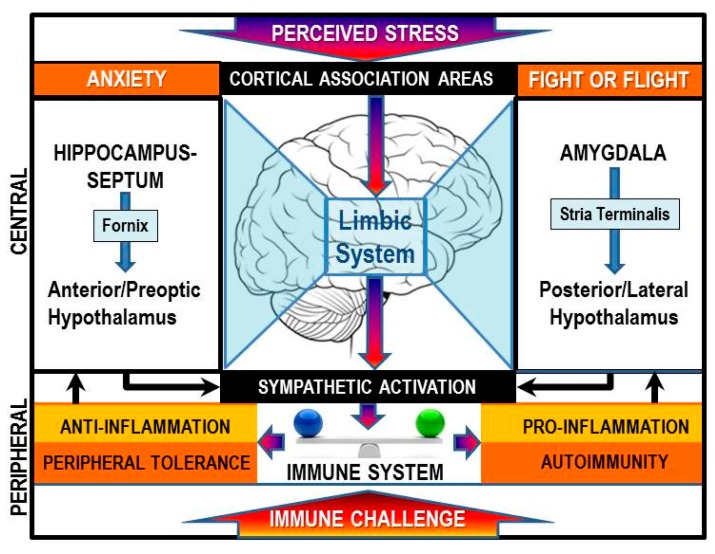
The brain perceives immune stimuli as a stressor via both humoral and via hardwired pathways to the central nervous system (CNS). This information, along with other psychosocial stressors is integrated by cortical association areas, the hypothalamus, and limbic circuits and provides a coordinated stress responses by the two major efferent stress pathways, one of which is the sympathetic nervous system (SNS). The SNS response influences the inflammatory state. Under conditions where the antigen is not eliminated and there is a break in immune tolerance autoimmunity can be triggered.

**Figure 7:**
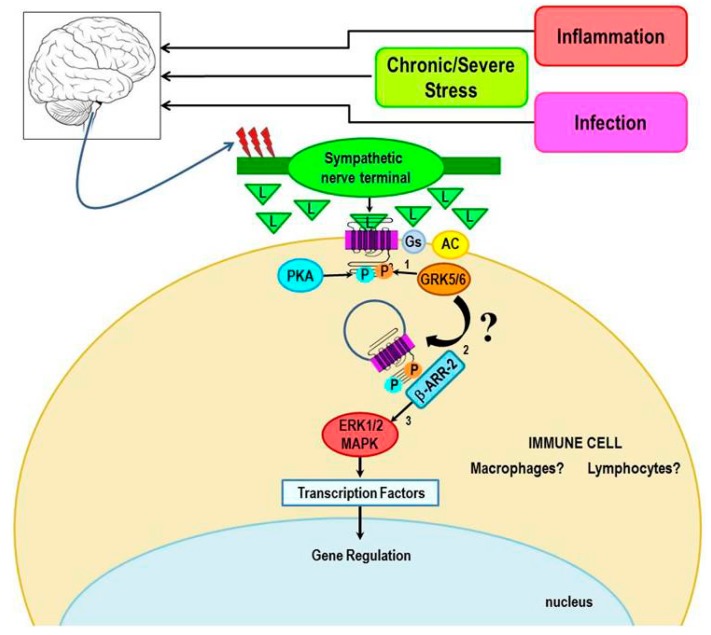
The hypothetical model for shifting β_2_-AR signaling from the canonical pathway towards the non-canonical pathway in immune cells is illustrated. We propose that signal transduction switching towards β-arrestin-mediated signaling occurs under conditions of unchecked immune cell activation or chronic or severe stress, both of which increase the firing rates of sympathetic nerves (red lightning bolts) and elevates local NE concentrations. These conditions favor the phosphorylation of the receptor by GRK 5/6 rather than GRK 2, thus promoting β-arrestin-2-mediated signaling via the MAPK, ERK1/2.

Chronically elevated SNS tone occurs in response to inflammatory conditions or immune challenge [102,103]. High circulating concentrations of tumor necrosis factor-α (TNF-α), interleukin (IL)-1 and IL-6 act at the hypothalamus to stimulate CNS pathways that drive elevated sympathetic nerve firing rates in relevant target tissues, including secondary lymphoid tissues and at sites of inflammation (Figure 6). Under acute inflammatory responses, sympathetic nerves increase their release of NE in secondary lymphoid tissues and at sites of inflammation. NE activates β_2_-ARs, which subsequently suppress cell-mediated immune responses and inflammation by activating the canonical pathway. This negative feedback circuit is important for immune response resolution and restoration of immune system homeostasis after pathogen clearance. However, whether there are physiologic or pathologic conditions in which β_2_-AR activation in immune cells induces activation of non-canonical pathway signaling is unclear. Studies using cultured kidney, fibroblast, glioblastoma cell lines and cardiac myocytes have shown that high agonist concentrations can shift β_2_-AR signaling towards the ERK/MAPK pathway. The extent to which β_2_-AR in cells of the immune system can signal via ERK under physiologic conditions such as during an inflammatory response, infection or stress, conditions which increase SNS activity and thus high agonist concentrations, are yet to be determined. If high agonist concentrations can shift β_2_-AR signaling toward ERK-mediated signaling in immune cells (Figure 7), it would have significant clinical implications for the impact of chronic stress on human health, particularly chronic inflammatory and cell-mediated diseases. In this concept paper, we provide examples from the literature, and our laboratory, that support signal-shifting by β_2_-ARs in cells of the immune system. Further, we describe physiologic and pathologic conditions and potential mechanisms responsible for β_2_-AR signal-switching from its canonical activation of cAMP to activation of the ERK pathway.

### 4.2. Evidence for Non-Canonical Signaling with Context-Dependent Inflammatory Responses

The SNS regulates the functions of both innate and adaptive immune cells. In *in vivo* and *in vitro* studies, stimulatory or inhibitory effects of NE are reported, depending upon the model system or tissue examined, type of adrenergic receptor activated (α-AR vs β_2_-AR), and the immune stimuli applied, the time point of evaluation after immune activation, and the environmental conditions of the study. However, most studies investigating the role of NE or β_2_-AR agonists on inflammation and cellular effector functions report immunosuppressive effects (reviewed in [1,2]). Still, many instances can be found in which, β_2_-AR stimulation of immune cells produces a seemingly paradoxical augmentation of inflammatory responses and cellular immunity. One explanation, at least for *in vitro* studies, is that the conditions of study may not reflect physiologic conditions. However, *in vivo* studies do provide support for β_2_-AR-induced enhancement of inflammatory and cell-mediated immune responses that is physiologically relevant in some diseases, and in thwarting pathogens.

**Figure 8:**
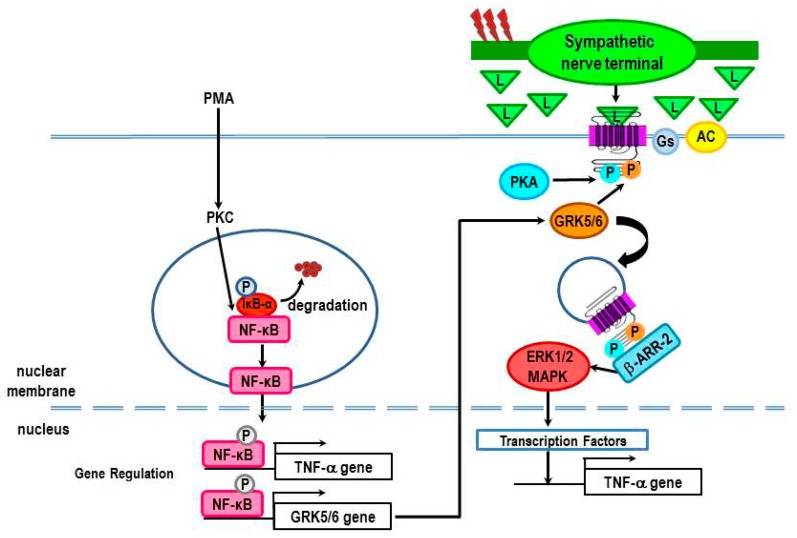
The presence of a β_2_-AR ligand during lipopolysaccharide (LPS) or phorbol 12-myristate 13-acetate (PMA)-induced macrophage activation causes reduced or increased production of TNF-α, IL-12 and nitric oxide respectively. The β_2_-AR-induced immunosuppression in LPS-treated macrophages is consistent with the activation of adenylyl cyclase (AC), and subsequently PKA. LPS promotes adenylyl cyclase activity, suppresses translocation of GRK2 to the membrane, and reduces the expression of GRKs 5 and 6. These LPS-induced effects are expected to increase β_2_-AR signaling via the cAMP-PKA canonical pathway. In contrast, the β_2_-AR-induced immune enhancement in PMA-treated macrophages is not consistent with receptor activation of the cAMP-PKA pathway. Instead, we propose that the responsible mechanism is a shift in receptor signaling from the canonical to the non-canonical pathway.

The immunomodulatory effect of β_2_-ARs is commonly studied as a co-stimulus in combination with a known immune activator. β_2_-AR activation of the canonical pathway can negatively or positively regulate the response of immune activators, such as concanavalin A, phytohemagglutinin, anti-CD3/anti-CD28, lipopolysaccharide (LPS) or phorbol 12-myristate 13-acetate (PMA), depending on the type of immune stimulus and timing of β_2_-AR stimulation relative to immune activation [104,105,106,107,108,109,110]. For example, the β_2_-agonist, isoproterenol (ISO) suppresses TNF-α production in LPS-stimulated peritoneal macrophages [104], consistent with canonical signaling and immunosuppressive effects of the SNS (Figure 8). Interestingly, β_2_-AR stimulation reduces ERK1/2 and p38 MAPK activity in the LPS-challenged macrophages [104]. In contrast, a few studies suggest that stimulation of β_2_-ARs in immune cells can enhance inflammation via MAPK signaling pathways (*i.e*., ERKs, p38, and JNKs) (Figure 8). For example, β_2_-AR stimulation is reported to enhance TNF-α, IL-12, and nitric oxide production in murine peritoneal macrophages, human peripheral monocytes, or human myelomonocyte leukemia cells (PLB-985) that are differentiated towards the macrophage lineage by treatment with phorbol 12-myristate 13-acetate (PMA) [104]. These findings are not easily explained by signaling via the canonical pathway; a few studies in non-immune cells may suggest some possible cAMP/PKA-mediated routes under specific conditions (reviewed in [105]). These are pathways that have not been clearly defined. More likely these findings may be explained by MAPK signaling. ISO increases TNF-α production in macrophages stimulated with PMA [104]. The β_2_-AR-induced TNF-α production is associated with an increase in ERK1/2 and p38 MAPKs expression [104], supporting activation of non-canonical β_2_-AR signaling pathways. In both of these conditions, pretreatment with the β-AR antagonist, propranolol blocks ISO-enhanced cytokine production, verifying mediation by β-ARs [104]. The mechanisms responsible for non-canonical pathway switching in PMA-treated macrophages are not clear. However, it is clear that the β_2_-AR signal transduction pathway activated depends on the immune stimulus. Thus, it seems that the β_2_-AR-mediated direction of change in inflammatory mediators produced by immune cells depends on the nature of the inflammatory stimulus, when the SNS is activated, and the context of the stimulation [104].

While the mechanisms that are responsible for different β_2_-AR functions are not known, we are hypothesizing that the different signaling pathways activated by LPS and PMA can lead to β_2_-AR modifications that alter β_2_-AR signaling (Figure 8). We propose that this cross-talk between mediators that activate monocytes/macrophages then results in an appropriate context-dependent β_2_-AR signaling response. While no studies have directly tested this hypothesis, published findings do lend support to this concept. LPS induces an inflammatory response via binding to the receptor-complex of CD14–toll-like receptor 4 (TLR4) expressed on macrophages. LPS stimulation of the TLR4 complex induces activation of both nuclear factor kappa-light-chain-enhancer of activated B cells (NF-κB) and MAPK pathways (ERK1 and 2, and p38) [111]. Activation of these pathways results in transcription of many genes that encode inflammatory mediators, including TNF-α, IL-12 and inducible nitric oxide synthase genes. More recently, LPS activation of these pathways has been shown to regulate many of the regulators that determine the functions of β_2_-ARs. LPS promotes adenylyl cyclase activity [112], suppresses the translocation of GRK2 to the membrane [113], and reduces the expression of GRKs 5 and 6 [114]. These effects are expected to increase β_2_-AR signaling via the cAMP-PKA canonical pathway (Figure 8). LPS-induced effects on GRK expression are mediated by NF-κB activation [114]. These findings provide a mechanism by which LPS, in addition to NE and epinephrine concentration-dependent regulation, can impact β_2_-AR functions.

Unfortunately, how phorbol esters, such as PMA, alter β_2_-ARs in a manner that causes them to stimulate, rather than inhibit TNF-α production remains unclear. Szenlenyi and coworkers [104] attribute the opposing modulatory effects of β_2_-AR stimulation on LPS and PMA -induced TNF-α production to differences in the kinetics of MAPK activation by the immune stimulus. Activation of MAPK occurs more rapidly and robustly after treatment with PMA than with LPS. In addition, LPS treatment also induces a more sustained activation of ERK and p38 than PMA. How differences in the kinetics and/or magnitude of MAPK (pERK and p38) production result in the opposing β_2_-AR agonist induced effects on TNF-α production, and the extent that this mechanism is responsible for the difference in receptor function, remains unclear. Alternatively PMA is also an activator of protein kinase C (PKC). In other non-immune cell types, PKC modulates β_2_-AR functions in several ways. Like PKA, this kinase can directly phosphorylate the β_2_-AR to promote receptor desensitization, albeit with less efficacy than PKA [27,115,116]. Further, PMA-induced PKC impairs adenylyl cyclase activity in prostate cells stimulated with a β-AR agonist [117], an effect expected to reduce cAMP and thus, PKA production. PKC also inhibits the activity of GRK2 and promotes GRK2 translocation to the membrane [118,119,120], effects expected to promote receptor desensitization. PMA-induced PKC also rapidly phosphorylates GRK5 in the simian fibroblast-like COS-1 (an abbreviation for CV-1 in Origin with SV40 genes) cell line, an event that reduces GRK5 activity [121]. However, these PMA-mediated changes in kinases that regulate β_2_-AR signaling either promote or reduce β_2_-AR signaling via cAMP. They do not explain the β_2_-AR-induced increase in TNF-α production in PMA-activated macrophages.

**Figure 9:**
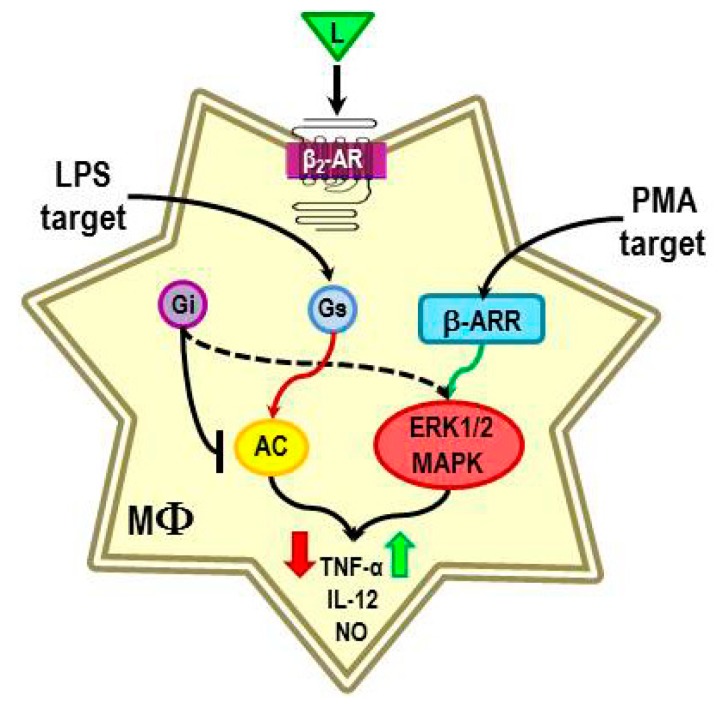
A possible mechanism for β_2_-AR-induced increases in TNF-α production in PMA-treated macrophages is by increasing gene transcription of GRK 5 or 6. PMA induces an increase in protein kinase C (PKC), which activates NF-κB by phosphorylating IκBα. Phosphorylation of IκBα releases NF-κB for nuclear translocation, and subsequent DNA binding. NF-κB increases gene expression of both TNF-α and GRK5. Increased GRK5 promotes signaling of β_2_-ARs in a β-arrestin-dependent manner, leading to activation of ERK1/2. ERK1/2 activates transcription factors that further promote TNF-α gene transcription to increase the production of TNF-α.

One possible mechanism for β_2_-AR potentiation of PMA-induced macrophage production of TNF-α is by regulating NF-κB (Figure 9). NF-κB is an inducible transcription factor that regulates gene expression of several inflammatory cytokines, including TNF-α [122]. Recently it was reported that PMA can phosphorylate IκBα, disinhibiting NF-κB nuclear translocation, and subsequent DNA binding [123,124]. Interestingly, a potential NF-κB binding site is present in the promoter region of GRK5. PMA increases the levels of GRK5 in myocytes [125], whereas treatment of these cells with an inhibitor of either NF-κB, or inhibitor of kappa B (IκB) kinase 2 decreases GRK5 [125]. These findings provide a potential mechanism to explain increases in TNF-α production by PMA-differentiated monocytes in the presence of a β_2_-AR agonist [104]. A PMA-induced increase in GRK5 could result in β_2_-AR phosphorylation by GRK5, which would promote signaling via β-arrestin-mediated ERK1/2 activation, and thus amplify TNF-α production. This hypothesis, as well as, GRK5–mediated β-arrestin recruitment to the β_2_-AR, should be examined in future studies.

Another study provides supports for β_2_-AR-β-arrestin-dependent signaling in macrophages. Tan *et al*. [126] report that salmeterol, a longer-lasting β_2_-AR agonist than ISO, increases IL-1β and IL-6 mRNA and protein levels in a number of unstimulated murine and human monocyte and macrophage cell lines. The effect of salmeterol on cytokine transcription was not mediated by PKA, but could be completely blocked by inhibitors of either the ERK1/2 and p38 MAPK pathway. These findings are consistent with other observations that identify salmeterol as a β_2_-AR agonist with β-arrestin “biased” signaling [127,128].

### 4.3. Evidence for β_2_-AR Non-Canonical Signaling in Adaptive Immune Responses

The possibility of β_2_-AR signaling via both cAMP-PKA and β-arrestin also extends to cells of adaptive immunity. The role of the SNS in regulating T lymphocyte functions has been best studied in CD4+ T cells, which orchestrate many aspects of adaptive immune responses, particularly for promoting either a cellular or humoral immune response. Naïve CD4+ T (T helper (Th) 0) cells can differentiate into either Th1 or Th2 cells, which drive cellular or humoral immune responses, respectively [129]. The SNS regulates the differentiation of Th0 cells to Th1 and Th2 cells; however, the latter occurs indirectly via effects on cytokine production by Th1 cells that suppress Th2 cell differentiation. This is consistent with the expression of β_2_-AR on Th0 and Th1 cells and their absence on Th2 cells. Cellular immune responses are directed by Th1 cells through their production of IL-2, which enhance proliferation and expansion of activated naïve CD4+ T cells and their production of interferon (IFN)-γ that promote Th1 cell but suppresses Th2 cell differentiation. Treatment of cultured naïve CD4+ T lymphocytes with NE or selective β_2_-AR agonists reduces IL-2 and IFN-γ (reviewed in [129]). This limits the ability of Th0 cells to proliferate and to generate CD4+ Th1 cells. These findings are consistent with β_2_-AR activation of cAMP and the general view that the SNS suppresses cellular immunity.

When naïve CD4+ Th0 cells are activated by an antigen or by anti-CD3/CD28 costimulation with or without NE or a β_2_-AR agonist, β_2_-AR stimulation increases Th0 cell production of IFN-γ [130]. In this scenario, the increased production of IFN-γ enhances Th1 cell proliferation and differentiation, promoting rather than inhibiting cellular immunity [130]. So, stimulation of β_2_-ARs prior to antigen activation decreases IFN-γ synthesis, while β_2_-agonist stimulation after antigen stimulation increases IFN-γ production. Despite the fact that these findings are over a decade old, the mechanisms by which β_2_-ARs induce activation of signaling pathways that increase in IFN-γ remains a mystery.

**Figure 10:**
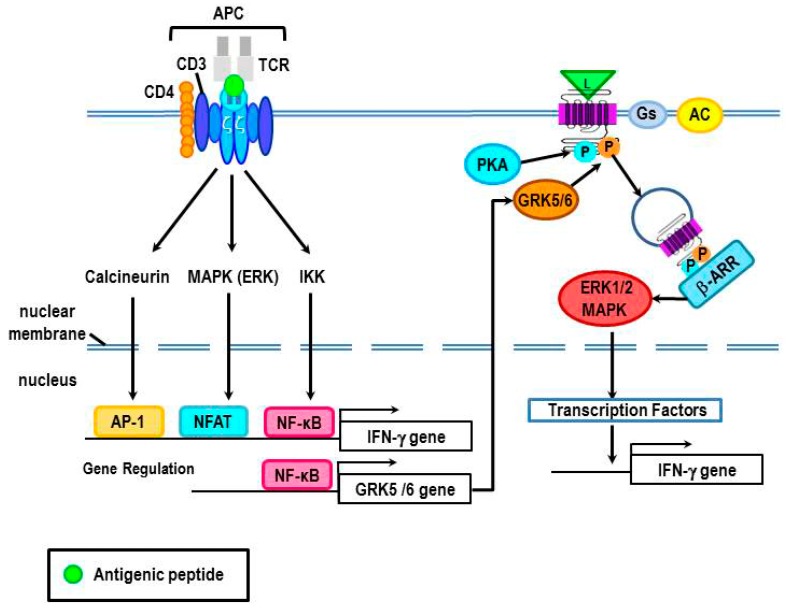
A proposed mechanism for β_2_-AR-mediated increases in IFN-γ in Th0 cells after antigen challenge is illustrated. T cell receptor (TCR) signal transduction is induced by the recognition of the antigen that is presented to the T cell by an antigen-presenting cell (APC). Antigen presentation can induce TCR activation of the signal transduction pathways, calcineurin, MAPK (ERK), and/or IKK, each with specific downstream signal cascades that lead to nuclear translocation of activator protein 1 (AP-1), nuclear factor of activated T cells (NFAT) and/or NF-κB, respectively. These transcription factors regulate gene transcription of cytokines, like IFN-γ. Additionally, NF-κB can up-regulate gene expression of GRK5/6, which can subsequently phosphorylate the activated β_2_-AR. GRK5/6 phosphorylation of the β_2_-AR recruits β-arrestin to the receptor leading to β-arrestin-mediated receptor desensitization, and possibly the activation of ERK1/2 MAPK. ERK1/2 is proposed to drive greater gene expression of IFN-γ.

Clues to the underlying mechanisms for β_2_-AR-mediated increases in IFN-γ may be gained from the Th0 cell activation conditions in which this occurs. Antigen activation of T cell receptors (TCRs) on Th0 cells induces synthesis of IFN-γ by activating the p38 MAPK pathway [131,132] (Figure 10). Interestingly, antigen stimulation of TCRs activates many intracellular signal pathways, including MAPKs (p38, ERK, and JNK), PKC, and increased intracellular calcium. These pathways activate the intracellular transcription factors, activating transcription factor 2 (ATF-2), nuclear factor of activated T cells (NFAT), activator protein 1 (AP-1), and NF-κB. As indicated previously, a potential NF-κB binding site is present in the promoter region of GRK5, at least in myocytes [125]. If this is true for CD4+ Th0 cells, this could provide a mechanism to explain the observed increase in IFN-γ production when Th0 cells are treated with a β_2_-AR agonist after antigen challenge. If so, antigen challenge could result in an increase in GRK5 expression and subsequent GRK5 phosphorylation of β_2_-ARs to induce G protein-independent signaling via β-arrestin to increase production of IFN-γ. Obviously, future studies are needed to test this hypothesis.

**Figure 11:**
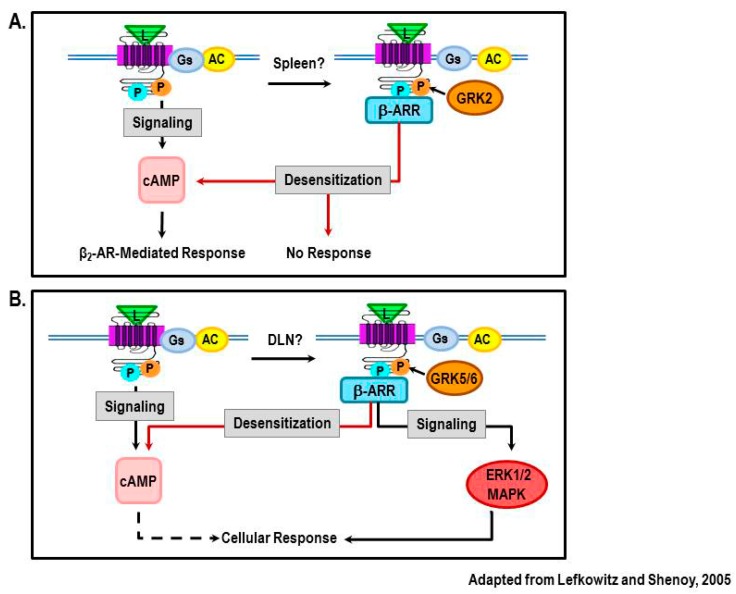
Findings from our laboratory using an animal model of the autoimmune disease, RA, reveal disease-related changes in β_2_-AR signaling that are dependent upon the lymphoid tissue examined. Here, we illustrate our proposed model to explain the different receptor signaling in spleen (**A**) and lymph nodes that drain the arthritic hind limbs (DLN) (**B**). (**A**) In the spleen, classical signaling via cAMP-PKA (shown on left side) is abolished and β_2_-AR agonists fail to inhibit the production of IFN-γ. High SNS tone caused by high levels of circulating inflammatory cytokines, results in PKA and GRK2 phosphorylation of β_2_-ARs in Th1 cells (shown on right side). Phosphorylation by PKA and GRK2 then induces receptor desensitization, thus β_2_-AR agonists fail to decrease IFN-γ production. For comparison, the canonical signaling pathway for β_2_-ARs is shown on the left. (**B**) In the DLN of arthritic rats, β_2_-AR agonists increase the production of IFN-γ. This finding supports a shift in receptor signaling away from the classical pathway (left side) towards the non-canonical pathway (right side). In the proposed model, high SNS nerve firing in the DLN coupled with high local levels of inflammatory cytokines induces β_2_-AR phosphorylation by PKA and is phosphorylated byGRK5/6. Phosphorylation by GRK5/6 leads to the recruitment of β-arrestin-2 to the receptor. Beta-arrestin-2 then switches receptor signaling to a β-arrestin-dependent activation of ERK1/2. ERK1/2 is well known to promote IFN-γ production.

Recent findings from our laboratory provide evidence for a shift in β_2_-AR signaling from cAMP to MAPK pathways in lymphocytes from rats with AA, a model of RA [46,133]. In these studies, β_2_-AR agonist treatment was started at disease onset and continued until severe disease. The lymph nodes that drain (DLNs) the arthritic joints and the spleens were harvested to assess sites where arthritogenic T cells develop. Mesenteric lymph nodes (MLNs) were examined as a site where T lymphocytes that do not transfer the disease to naïve animals reside. Interestingly, arthritic animals treated with the β_2_-AR agonist, terbutaline altered IFN-γ production differently in each lymphoid organ examined, increasing IFN-γ in DLN cells, having no effect on IFN-γ in splenocytes, and inhibiting IFN-γ in MLN cells [133]. Reduced IFN-γ concentrations in MLN cells is consistent with the well-known ability of β_2_-AR stimulation to reduce IFN-γ production by increasing cAMP levels. Findings in the spleen indicate β_2_-AR desensitization and down-regulation induced by chronic agonist stimulation as β-AR density and cAMP production are reduced in splenocytes from these rats [46] (illustrated in Figure 11A). However, the β_2_-AR-mediated increases in IFN-γ observed in DLN cells cannot be explained by the canonical signaling pathway for β_2_-ARs or a loss of receptor numbers or activity.

One possible explanation for the β_2_-AR agonist-induced increase in IFN-γ is a switch from cAMP-PKA signaling to the ERK-MAPK pathway, a pathway which is well known to increase IFN-γ [131,132] (Figure 11B). Support for this hypothesis is found from assessing phosphorylation of β_2_-ARs by PKA and GRK in DLN cells from adjuvant-induced arthritic rats [46]. Phosphorylation of β_2_-AR by PKA rises during severe disease, but falls during chronic disease, whereas phosphorylation of β_2_-AR by GRKs increases during both stages of disease [46]. This pattern is consistent with a GRK-dominant role in receptor signal transduction. GRK-coupled β_2_-AR signaling linked to elevated IFN-γ production in the DLNs is consistent with GRK-induced β-arrestin-mediated signaling via ERK pathways. Future studies are required to determine if this response is mediated by GRK5 or 6-induced β_2_-AR phosphorylation, recruitment of β-arrestin-2 to the receptor and increased production of ERK1/2. Interestingly, a similar β_2_-AR phosphorylation pattern in DLN cells occurs in rats challenged with the mycobacterial cell wall suspended in saline, indicating that pattern recognition receptors (*i.e*., toll-like receptors) are important for GRK-mediated β_2_-AR phosphorylation in DLN cells.

In contrast to our findings in DLN cells, elevated β_2_-AR phosphorylation by GRK in splenocytes was restricted to the chronic disease phase [46]. This, coupled with the inability of β_2_-AR agonists to increase cAMP or to alter IFN-γ, is consistent with PKA and GRK2-phosphorylation of the receptor, recruitment of β-arrestin1 and subsequent desensitization (Figure 11A). Future studies are needed to determine if GRK2 or 6 and ERK1/2 are elevated differentially in DLN and spleen cells during severe and chronic disease, and whether β_2_-agonist-induced increases in IFN-γ in DLN cells can be blocked by inhibitors of the ERK1/2 pathway.

It is intriguing that β_2_-ARs function differently in DLN and spleen cells in the adjuvant-induced arthritis model of RA. One possible explanation to be pursued in future studies is the possibility that the local environment of the DLN and spleen, with regard to the concentration and kinds of inflammatory mediators, differs dramatically. The DLN receive lymphatic drainage from the inflamed arthritic joints, whereas the spleen filters the blood. Thus, the concentrations of inflammatory cytokines, such as IL-1β and TNF-α are likely to be much greater in DLN than in the spleen, as these mediators would be diluted after entering the blood. The activity and levels of GRKs are dynamically influenced by inflammatory mediators (reviewed in [134]). Given that inflammatory mediators can induce expression of GRK2 or GRK5/6 depending upon the mediator, and that the different GRKs regulate receptor function (reviewed in [4]), the differences in β_2_-AR sensitivity, signaling and down-regulation between different immune organs could be due to differences in the local inflammatory environment.

Dramatic changes in the expression of GRK2 and 6 in peripheral blood mononuclear cells are observed in RA patients [83,84,85] and in immune organs from rats with experimental adjuvant-induced arthritis [135]. However, GRKs were not examined in DLNs of adjuvant-induced arthritic rats in the latter study. Interestingly, changes in GRKs were only observed in immune cells subsets that are critically involved in disease pathology, CD4+ T cells and B cells, but not in CD8+ T cells [135]. Similar down-regulation of GRK2 and GRK6 expression is reported in in PBMC of patients with MS [84,85] and in immune organs from rats with chronic relapsing EAE [84].

What are the implications of altered SNS signaling via β_2_-ARs in immune cells? The SNS regulates all aspects of innate and adaptive immune functions as part of its major role in integrating body functions to maintain homeostasis [1]. Cross-talk between these two regulatory systems is essential, and takes place in part via circulating cytokines acting at the hypothalamus, as well as, locally activated sensory nerves at inflamed sites. Activation of these afferent pathways induces greater activation of efferent sympathetic nerves in immune organs and the inflammatory sites. The immune activating stimulus in conjunction with the change in activity of sympathetic nerves and NE availability collectively direct how β_2_-ARs signal intracellularly. Data support that inflammatory cytokines, regulate both β_2_-AR expression and signal transduction by increasing the expression of intracellular GRKs. The exciting discovery that β_2_-AR can activate ERK1/2 by a Gs-independent, β-arrestin-dependent pathway raises many questions regarding SNS regulation of innate and adaptive immune functions in health and disease. Whether such signaling occurs in immune cells has not been determined. However, there are many cases in the literature where β_2_-ARs induce changes in cytokine responses in directions not explained by increases in cAMP, and that are blocked by β_2_-AR antagonists. Such responses could be explained via β_2_-AR-induced increases in ERK1/2. It is also unclear if this alternate signaling pathway represents normal physiological regulation of immune functions that are adaptive or are strictly pathologic. There is data to support the latter, as GRK6 expression, which would drive β_2_-AR signaling via ERK, is increased in MRL/lpr mice, a murine model of systemic lupus erythematosus (SLE), and in spleens upon autopsies of SLE patients [136]. Further, loss of GRK6 in knockout mice is associated with altered functions of other GPCRs that contribute to tumor progression [137], hyperalgesia [138,139], and Parkinson’s disease [140]. Chronic and severe stressors that elevate SNS activity, a condition that seems to be required to shift β_2_-AR signaling, are linked to many diseases. Thus, it will be important to understand how β_2_-AR signaling via ERK pathways is involved in susceptibility, development and progression of diseases mediated by the immune system.

## Abbreviations

5'-AMP: 5'-monophosphate
β-ARR-2: β-arrestin 2
pβ_2_-AR_PKA_: β_2_-AR phosphorylation by PKA
ATF-2: activating transcription factor 2
AC: activator protein 1 (AP-1, adenylate cyclase
ARs: adrenergic receptors
AKAPs: A-kinase anchor proteins
APC: antigen-presenting cell
C: catalytic subunit
CREB: cAMP response element-binding
CNS: central nervous system
JNK: c-Jun *N*-terminal kinase
DLNs: draining lymph nodes (of arthritic joints)
Epac: exchange protein directly activated by cAMP
EAE: experimental autoimmune encephalomyelitis
ERK: extracellular signal-regulated protein kinase
Gα_s_: G protein alpha s subunit
Gβγ: G protein beta/gamma subunit
GRKs: G protein-coupled kinases
GDP: guanosine diphosphate
GTP: guanosine triphosphate
GPCRs: G protein-coupled receptors
HEK: human embryonic kidney
IκBα: inhibitor of kappa B
IFN: interferon
IL: interleukin
cAMP_i_: intracellular cAMP
ISO: isoproterenol
L: ligand
LPS: lipopolysaccharide
MLNs: mesenteric lymph nodes
MAPK: mitogen-activated protein kinase
MS: multiple sclerosis
NE: norepinephrine
NF-κB: nuclear factor kappa-light-chain-enhancer of activated B cells
NFAT: nuclear factor of activated T cells
PBMCs: peripheral blood mononuclear cells
PMA: phorbol 12-myristate 13-acetate
PDE: phosphodiesterases
PKA: protein kinase A
PKC: protein kinase C
TNF-α: tumor necrosis factor-α
R: regulatory subunit dimer
RA: rheumatoid arthritis
SNS: sympathetic nervous system
TCRs: T cell receptors
Th: T helper

## References

[1] Bellinger D.L, Lorton D. (2014). Autonomic regulation of cellular immune function. Auton. Neurosci..

[2] Nance D.M., Sanders V.M. (2007). Autonomic innervation and regulation of the immune system (1987–2007). Brain Behav. Immun..

[3] Sivertsen B., Holliday N., Madsen A.N., Holst B. (2013). Functionally biased signaling properties of 7TM receptors—Opportunities for drug development for the ghrelin receptor. Br. J. Pharmacol..

[4] Gurevich E.V., Tesmer J.J., Mushegian A., Gurevich V.V. (2012). G protein-coupled receptor kinases: More than just kinases and not only for GPCRs. Pharmacol. Ther..

[5] Shukla A.K., Xiao K., Lefkowitz R.J. (2011). Emerging paradigms of β-arrestin-dependent seven transmembrane receptor signaling. Trends Biochem. Sci..

[6] Schulte G., Levy F.O. (2007). Novel aspects of G-protein-coupled receptor signaling—Different ways to achieve specificity. Acta Physiol. (Oxf.).

[7] Dessauer C.W. (2009). Adenylyl cyclase–A-kinase anchoring protein complexes: The next dimension in cAMP signaling. Mol. Pharmacol..

[8] Shaw A.S. (2006). Lipid rafts: Now you see them, now you don’t. Nat. Immunol..

[9] Simons K., Ikonen E. (1997). Functional rafts in cell membranes. Nature.

[10] Lefkowitz R.J. (2007). Seven transmembrane receptors: Something old, something new. Acta. Physiol. (Oxf.).

[11] Vandamme J., Castermans D., Thevelein J.M. (2012). Molecular mechanisms of feedback inhibition of protein kinase A on intracellular cAMP accumulation. Cell Signal..

[12] Kohm A.P., Sanders V.M. (2001). Norepinephrine and β_2_-adrenergic receptor stimulation regulate CD4+ T and B lymphocyte function *in vitro* and *in vivo*. Pharmacol. Rev..

[13] Baillie G.S., Houslay M.D. (2005). Arrestin times for compartmentalised cAMP signalling and phosphodiesterase-4 enzymes. Curr. Opin. Cell Biol..

[14] Shirshev S.V. (2011). Role of Epac proteins in mechanisms of cAMP-dependent immunoregulation. Biochemistry (Mosc.).

[15] Duan B., Davis R., Sadat E.L., Collins J., Sternweis P.C., Yuan D., Jiang L.I. (2010). Distinct roles of adenylyl cyclase VII in regulating the immune responses in mice. J. Immunol..

[16] Smith K.E., Gu C., Fagan K.A., Hu B., Cooper D.M. (2002). Residence of adenylyl cyclase type 8 in caveolae is necessary but not sufficient for regulation by capacitative Ca^2+^ entry. J. Biol. Chem..

[17] Pontier S.M., Percherancier Y., Galandrin S., Breit A., Galés C., Bouvier M. (2008). Cholesterol-dependent separation of the β_2_-adrenergic receptor from its partners determines signaling efficacy: Insight into nanoscale organization of signal transduction. J. Biol. Chem..

[18] Hertz A.L., Beavo J.A. (2011). Cyclic nucleotides and phosphodiesterases in monocytic differentiation. Handb. Exp. Pharmacol..

[19] Griffiths G., Hollinshead R., Hemmings B.A., Nigg E.A. (1990). Ultrastructural localization of the regulatory (RII) subunit of cyclic AMP-dependent protein kinase to subcellular compartments active in endocytosis and recycling of membrane receptors. J. Cell Sci..

[20] Serezani C.H., Ballinger M.N., Aronoff D.M., Peters-Golden M. (2008). Cyclic AMP: Master regulator of innate immune cell function. Am. J. Respir. Cell Mol. Biol..

[21] Chin K.V., Yang W.L., Ravatn R., Kita T., Reitman E., Vettori D., Cvijic M.E., Shin M., Iacono L. (2002). Reinventing the wheel of cyclic AMP: Novel mechanisms of cAMP signaling. Ann. N. Y. Acad. Sci..

[22] Schillace R.V., Andrews S.F., Galligan S.G., Burton K.A., Starks H.J., Bouwer H.G., McKnight G.S., Davey M.P., Carr D.W. (2005). The role of protein kinase a anchoring via the RIIα regulatory subunit in the murine immune system. J. Immunol..

[23] Skålhegg B.S., Taskén K., Hansson V., Huitfeldt H.S., Jahnsen T., Lea T. (1994). Location of cAMP-dependent protein kinase type I with the TCR-CD3 complex. Science.

[24] Skålhegg B.S., Tasken K. (2000). Specificity in the cAMP/PKA signaling pathway. Differential expression, regulation, and subcellular localization of subunits of PKA. Front. Biosci..

[25] Clark R.B., Knoll B.J., Barber R. (1999). Partial agonists and G protein-coupled receptor desensitization. Trends Pharmacol. Sci..

[26] Rockman H.A., Koch W.J., Lefkowitz R.J. (2002). Seven-transmembrane-spanning receptors and heart function. Nature.

[27] Tran T.M., Friedman J., Qunaibi E., Baameur F., Moore R.H., Clark R.B. (2004). Characterization of agonist stimulation of cAMP-dependent protein kinase and G protein-coupled receptor kinase phosphorylation of the β_2_-adrenergic receptor using phosphoserine-specific antibodies. Mol. Pharmacol..

[28] Hausdorff W.P., Bouvier M., O’Dowd B.F., Irons G.P., Caron M.G., Lefkowitz R.J. (1989). Phosphorylation sites on two domains of the β_2_-adrenergic receptor are involved in distinct pathways of receptor desensitization. J. Biol. Chem..

[29] Lohse M.J., Benovic J.L., Codina J., Caron M.G., Lefkowitz R.J. (1990). Beta-arrestin: A protein that regulates β-adrenergic receptor function. Science.

[30] Lefkowitz R.J. (1998). G protein-coupled receptors III. New roles for receptor kinases and β-arrestins in receptor signaling and desensitization. J. Biol. Chem..

[31] Lefkowitz R.J., Shenoy S.K. (2005). Transduction of receptor signals by β-arrestins. Science.

[32] Pitcher J.A., Freedman N.J., Lefkowitz R.J. (1998). G protein-coupled receptor kinases. Annu. Rev. Biochem..

[33] Benovic J.L., Kuhn H., Weyand I., Codina J., Caron M.G., Lefkowitz R.J. (1987). Functional desensitization of the isolated beta-adrenergic receptor by the β-adrenergic receptor kinase: Potential role of an analog of the retinal protein arrestin (48-kDa protein). Proc. Natl. Acad. Sci. USA.

[34] Hausdorff W.P., Lohse M.J., Bouvier M., Liggett S.B., Caron M.G., Lefkowitz R.J. (1990). Two kinases mediate agonist-dependent phosphorylation and desensitization of the β_2_-adrenergic receptor. Symp. Soc. Exp. Biol..

[35] Inglese J., Freedman N.J., Koch W.J., Lefkowitz R.J. (1993). Structure and mechanism of the G protein-coupled receptor kinases. J. Biol. Chem..

[36] Freedman N.J., Lefkowitz R.J. (1996). Desensitization of G protein-coupled receptors. Recent Prog. Horm. Res..

[37] Krupnick J.G., Benovic J.L. (1998). The role of receptor kinases and arrestins in G protein-coupled receptor regulation. Annu. Rev. Pharmacol. Toxicol..

[38] Ferguson S.S., Ménard L., Barak L.S., Koch W.J., Colapietro A.M., Caron M.G. (1995). Role of phosphorylation in agonist-promoted β_2_-adrenergic receptor sequestration. Rescue of a sequestration-defective mutant receptor by βARK1. J. Biol. Chem..

[39] Reiter E., Lefkowitz R.J. (2006). GRKs and beta-arrestins: Roles in receptor silencing, trafficking and signaling. Trends Endocrinol. Metab..

[40] Goodman O.B., Krupnick J.G., Santini F., Gurevich V.V., Penn R.B., Gagnon A.W., Keen J.H., Benovic J.L. (1996). β-arrestin acts as a clathrin adaptor in endocytosis of the β_2_-adrenergic receptor. Nature.

[41] Rockman H.A., Chien K.R., Choi D.J., Iaccarino G., Hunter J.J., Ross J., Lefkowitz R.J., Koch W.J. (1998). Expression of a β-adrenergic receptor kinase 1 inhibitor prevents the development of myocardial failure in gene-targeted mice. Proc. Natl. Acad. Sci. USA..

[42] Tachibana H., Naga Prasad S.V., Lefkowitz R.J., Koch W.J., Rockman H.A. (2005). Level of β-adrenergic receptor kinase 1 inhibition determines degree of cardiac dysfunction after chronic pressure overload-induced heart failure. Circulation.

[43] Lymperopoulos A., Rengo G., Funakoshi H., Eckhart A.D., Koch W.J. (2007). Adrenal GRK2 upregulation mediates sympathetic overdrive in heart failure. Nat. Med..

[44] Wang W.C., Mihlbachler K.A., Brunnett A.C., Liggett S.B. (2009). Targeted transgenesis reveals discrete attenuator functions of GRK and PKA in airway β_2_-adrenergic receptor physiologic signaling. Proc. Natl. Acad. Sci. USA.

[45] Balabanian K., Lagane B., Pablos J.L., Laurent L., Planchenault T., Verola O., Lebbe C., Kerob D., Dupuy A., Hermine O. (2005). WHIM syndromes with different genetic anomalies are accounted for by impaired CXCR4 desensitization to CXCL12. Blood.

[46] Lorton D., Bellinger D.L., Schaller J.A., Shewmaker E., Osredkar T., Lubahn C. (2013). Altered sympathetic-to-immune cell signaling via β_2_-adrenergic receptors in adjuvant arthritis. Clin. Dev. Immunol..

[47] Baillie G.S., Sood A., McPhee I., Gall I., Perry S.J., Lefkowitz R.J., Houslay M.D. (2003). β-Arrestin-mediated PDE4 cAMP phosphodiesterase recruitment regulates β-adrenoceptor switching from Gs to Gi. Proc. Natl. Acad. Sci. USA..

[48] Shenoy SK., Drake M.T., Nelson C.D., Houtz D.A., Xiao K., Madabushi S., Reiter E., Premont R.T., Lichtarge O., Lefkowitz R.J. (2006). Beta-arrestin-dependent, G protein-independent ERK1/2 activation by the β_2_-adrenergic receptor. J. Biol. Chem..

[49] Chang L., Karin M. (2001). Mammalian MAP kinase signalling cascades. Nature.

[50] Pearson G., Robinson F., Beers Gibson T., Xu B.E., Karandikar M., Berman K., Cobb M.H. (2001). Mitogen-activated protein (MAP) kinase pathways: Regulation and physiological functions. Endocr. Rev..

[51] Ip Y.T., Davis R.J. (1998). Signal transduction by the c-Jun *N*-terminal kinase (JNK)—From inflammation to development. Curr. Opin. Cell Biol..

[52] Yang J., New L., Jiang Y., Han J., Su B. (1998). Molecular cloning and characterization of a human protein kinase that specifically activates c-Jun *N*-terminal kinase. Gene.

[53] Arthur J.S., Ley S.C. (2013). Mitogen-activated protein kinases in innate immunity. Nat. Rev. Immunol..

[54] Ivashkiv L.B. (2011). Inflammatory signaling in macrophages: Transitions from acute to tolerant and alternative activation states. Eur. J. Immunol..

[55] Ashwell J.D. (2006). The many paths to p38 mitogen-activated protein kinase activation in the immune system. Nat. Rev. Immunol..

[56] Furler R.L, Uittenbogaart C.H. (2010). Signaling through the P38 and ERK pathways: A common link between HIV replication and the immune response. Immunol. Res..

[57] Zeiser R., Negrin R.S. (2008). Interleukin-2 receptor downstream events in regulatory T cells: Implications for the choice of immunosuppressive drug therapy. Cell Cycle.

[58] Benczik M., Gaffen S.L. (2004). The interleukin (IL)-2 family cytokines: Survival and proliferation signaling pathways in T lymphocytes. Immunol. Investig..

[59] Alberola-Ila J., Hernández-Hoyos G. (2003). The Ras/MAPK cascade and the control of positive selection. Immunol. Rev..

[60] Essayan D.M. (2001). Cyclic nucleotide phosphodiesterases. J. Allergy Clin. Immunol..

[61] Abrahamsen H., Baillie G., Ngai J., Vang T., Nika K., Ruppelt A., Mustelin T., Zaccolo M., Houslay M., Taskén K. (2004). TCR- and CD28-mediated recruitment of phosphodiesterase 4 to lipid rafts potentiates TCR signaling. J. Immunol..

[62] Bjørgo E., Solheim S.A., Abrahamsen H., Baillie G.S., Brown K.M., Berge T., Okkenhaug K., Houslay M.D., Taskén K. (2010). Cross talk between phosphatidylinositol 3-kinase and cyclic AMP (cAMP)-protein kinase a signaling pathways at the level of a protein kinase B/β-arrestin/cAMP phosphodiesterase 4 complex. Mol. Cell. Biol..

[63] Erdogan S., Houslay M.D. (1997). Challenge of human Jurkat T-cells with the adenylate cyclase activator forskolin elicits major changes in cAMP phosphodiesterase (PDE) expression by up-regulating PDE3 and inducing PDE4D1 and PDE4D2 splice variants as well as down-regulating a novel PDE4A splice variant. Biochem. J..

[64] Giembycz M.A. (1996). Phosphodiesterase 4 and tolerance to β_2_-adrenoceptor agonists in asthma. Trends Pharmacol. Sci..

[65] Francis S.H., Blount M.A., Corbin J.D. (2011). Mammalian cyclic nucleotide phosphodiesterases: Molecular mechanisms and physiological functions. Physiol. Rev..

[66] Mika D., Leroy J., Vandecasteele G., Fischmeister R. (2012). PDEs create local domains of cAMP signaling. J. Mol. Cell. Cardiol..

[67] Page C.P., Spina D. (2011). Phosphodiesterase inhibitors in the treatment of inflammatory diseases. Handb. Exp. Pharmacol..

[68] Chuang T.T., Sallese M., Ambrosini G., Parruti G., de Blasi A. (1992). High expression of β-adrenergic receptor kinase in peripheral human blood leukocytes. J. Biol. Chem..

[69] Loudon R.P., Perussia B., Benovic J.L. (1996). Differentially regulated expression of the G protein-coupled receptor kinases, βARK and GRK6, during myelomonocytic cell development *in vitro*. Blood.

[70] Oppermann M., Mack M., Proudfoot A.E., Olbrich H. (1999). Differential effects of CC chemokines on CC chemokine receptor 5 (CCR5) phosphorylation and identification of phosphorylation sites on the CCR5 carboxyl terminus. J. Biol. Chem..

[71] Peng Y.X., Shan J., Qi X.Y., Zhang S.J., Ma S.P., Wang N., Li J.P., Xue H., Wu M. (2006). The catecholamine-β-adrenoreceptor-cAMP system and prediction of cardiovascular events in hypertension. Clin. Exp. Pharmacol. Physiol..

[72] Bernardin G., Strosberg A.D., Bernard A., Mattei M., Marullo S. (1998). β-Adrenergic receptor-dependent and -independent stimulation of adenylate cyclase is impaired during severe sepsis in humans. Intensive Care Med..

[73] Singh M., Notterman D.A., Metakis L. (1993). Tumor necrosis factor produces homologous desensitization of lymphocyte β_2_-adrenergic responses. Circ. Shock.

[74] Silverman H.J., Lee N.H., el-Fakahany E.E. (1990). Effects of canine endotoxin shock on lymphocytic β-adrenergic receptors. Circ. Shock.

[75] Baerwald C., Graefe C., von Wichert P., Krause A. (1992). Decreased density of β-adrenergic receptors on peripheral blood mononuclear cells in patients with rheumatoid arthritis. J. Rheumatol..

[76] Baerwald C.G., Wahle M., Ulrichs T., Jonas D., von Bierbrauer A., von Wichert P., Burmester G.R., Krause A. (1999). Reduced catecholamine response of lymphocytes from patients with rheumatoid arthritis. Immunobiology.

[77] Wahle M., Hanefeld G., Brunn S., Straub R.H., Wagner U., Krause A., Häntzschel H., Baerwald C.G. (2006). Failure of catecholamines to shift T-cell cytokine responses toward a Th2 profile in patients with rheumatoid arthritis. Arthritis Res. Ther..

[78] Gao H., Xue Q., Lin Y., Wang L, Zhu G, Zhao Q, Chen Y. (2001). Role of β-adrenoceptor at different stages of bronchial asthma. Chin. Med. J. (Engl.).

[79] Hataoka I., Okayama M., Sugi M., Inoue H., Takishima T., Shirato K. (1993). Decrease in β-adrenergic receptors of lymphocytes in spontaneously occurring acute asthma. Chest.

[80] Iizuka K., Yoshie Y., Nakazawa T. (1991). Hormone-sensitive adenylate cyclase system in lymphocytes from asthmatic patients: Possible defects at the postreceptor sites. Ann. Allergy.

[81] Oyama N., Urasawa K., Kaneta S., Sakai H., Saito T., Takagi C., Yoshida I., Kitabatake A., Tsutsui H. (2005). Chronic β-adrenergic receptor stimulation enhances the expression of G-Protein coupled receptor kinases, GRK2 and GRK5, in both the heart and peripheral lymphocytes. Circ. J..

[82] Gros R., Benovic J.L., Tan C.M., Feldman R.D. (1997). G-protein-coupled receptor kinase activity is increased in hypertension. J. Clin. Investig..

[83] Lombardi M.S., Kavelaars A., Schedlowski M., Bijlsma J.W., Okihara K.L., van de Pol M., Ochsmann S., Pawlak C., Schmidt R.E., Heijnen C.J. (1999). Decreased expression and activity of G-protein-coupled receptor kinases in peripheral blood mononuclear cells of patients with rheumatoid arthritis. FASEB J..

[84] Vroon A., Kavelaars A., Limmroth V., Lombardi M.S., Goebel M.U., van dam A.M., Caron M.G., Schedlowski M., Heijnen C.J. (2005). G protein-coupled receptor kinase 2 in multiple sclerosis and experimental autoimmune encephalomyelitis. J. Immunol..

[85] Giorelli M., Livrea P., Trojano M. (2004). Post-receptorial mechanisms underlie functional disregulation of β_2_-adrenergic receptors in lymphocytes from Multiple Sclerosis patients. J. Neuroimmunol..

[86] Zoukos Y., Thomaides T.N., Kidd D., Cuzner M.L., Thompson A. (2003). Expression of β_2_-adrenoreceptors on peripheral blood mononuclear cells in patients with primary and secondary progressive multiple sclerosis: A longitudinal six month study. J. Neurol. Neurosurg. Psychiatry.

[87] Zoukos Y., Kidd D., Woodroofe M.N., Kendall B.E., Thompson A.J., Cuzner M.L. (1994). Increased expression of high affinity IL-2 receptors and beta-adrenoceptors on peripheral blood mononuclear cells is associated with clinical and MRI activity in multiple sclerosis. Brain.

[88] DeWire S.M., Ahn S., Lefkowitz R.J., Shenoy S.K. (2007). β-arrestins and cell signaling. Annu. Rev. Physiol..

[89] Luttrell L.M., Gesty-Palmer D. (2010). Beyond desensitization: Physiological relevance of arrestin-dependent signaling. Pharmacol. Rev..

[90] Watari K., Nakaya M., Kurose H. (2014). Multiple functions of G protein-coupled receptor kinases. J. Mol. Signal..

[91] Nobles K.N., Xiao K., Ahn S., Shukla A.K., Lam C.M., Rajagopal S., Strachan R.T., Huang T.Y., Bressler E.A., Hara M.R. (2011). Distinct phosphorylation sites on the β_2_-adrenergic receptor establish a barcode that encodes differential functions of β-arrestin. Sci. Signal..

[92] Trester-Zedlitz M., Burlingame A., Kobilka B., von Zastrow M. (2005). Mass spectrometric analysis of agonist effects on posttranslational modifications of the β_2_-adrenoceptor in mammalian cells. Biochemistry.

[93] Millman E.E., Rosenfeld J.L., Vaughan D.J., Nguyen J., Dai W., Alpizar-Foster E., Clark R.B., Knoll B.J., Moore R.H. (2004). Endosome sorting of β_2_-adrenoceptors is GRK5 independent. Brit. J. Pharmacol..

[94] Pierce K.L., Lefkowitz R.J. (2001). Classical and new roles of β-arrestins in the regulation of G-protein-coupled receptors. Nat. Rev. Neurosci..

[95] Claing A., LaPorte S.A., Caron M.G., Lefkowitz R.J. (2002). Endocytosis of G protein-coupled receptors: Roles of G protein-coupled receptor kinases and beta-arrestin proteins. Prog. Neurobiol..

[96] McDonald P.H., Chow C.W., Miller W.E., LaPorte S.A., Field M.E., Lin F.T., Davis R.J., Lefkowitz R.J. (2000). β-arrestin 2: A receptor-regulated MAPK scaffold for the activation of JNK3. Science.

[97] Luttrell L.M., Roudabush F.L., Choy E.W., Miller W.E., Field M.E., Pierce K.L., Lefkowitz R.J. (2001). Activation and targeting of extracellular signal-regulated kinases by β-arrestin scaffolds. Proc. Natl. Acad. Sci. USA.

[98] Luttrell L.M., Ferguson S.S., Daaka Y., Miller W.E., Maudsley S., Della Rocca G.J., Lin F., Kawakatsu H., Owada K., Luttrell D.K. (1999). β-Arrestin-dependent formation of β_2_ adrenergic receptor-Src protein kinase complexes. Science.

[99] Sun Y., Cheng Z., Ma L., Pei G. (2002). β-Arrestin2 is critically involved in CXCR4-mediated chemotaxis, and this is mediated by its enhancement of p38 MAPK activation. J. Biol. Chem..

[100] Kim K.S., Park D.H., Wessel T.C., Song B., Wagner J.A., Joh T.H. (1993). A dual role for the cAMP-dependent protein kinase in tyrosine hydroxylase gene expression. Proc. Natl. Acad. Sci. USA.

[101] Pitcher J., Lohse M.J., Codina J., Caron M.G., Lefkowitz R.J. (1992). Desensitization of the isolated β_2_-adrenergic receptor by beta-adrenergic receptor kinase, cAMP-dependent protein kinase, and protein kinase C occurs via distinct molecular mechanisms. Biochemistry.

[102] Meltzer J.C., MacNeil B.J., Sanders V., Pylypas S., Jansen A.H., Greenberg A.H., Nance D.M. (2003). Contribution of the adrenal glands and splenic nerve to LPS-induced splenic cytokine production in the rat. Brain Behav. Immun..

[103] MacNeil B.J., Jansen A.H., Greenberg A.H., Nance D.M. (1996). Activation and selectivity of splenic sympathetic nerve electrical activity response to bacterial endotoxin. Am. J. Physiol..

[104] Szelenyi J., Selmeczy Z., Brozik A., Medgyesi D., Magocsi M. (2006). Dual β-adrenergic modulation in the immune system. Stimulus-dependent effect of isoproterenol on MAPK activation and inflammatory mediator production in macrophages. Neurochem. Int..

[105] Gerlo S., Kooijman R., Beck I.M., Kolmus K., Spooren A., Haegeman G. (2011). Cyclic AMP: A selective modulator of NF-κB action. Cell. Mol. Life Sci..

[106] Elenkov I.J., Hasko G., Kovacs K.J., Vizi E.S. (1995). Modulation of lipopolysaccharide-induced tumor necrosis factor-α production by selective α- and β-adrenergic drugs in mice. J. Neuroimmunol..

[107] Ramer-Quinn D.S., Swanson M.A., Lee W.T., Sanders V.M. (2000). Cytokine production by naive and primary effector CD4+ T cells exposed to norepinephrine. Brain Behav. Immun..

[108] Van Oosterhout A.J., Stam W.B., Vanderschueren R.G., Nijkamp F.P. (1992). Effects of cytokines on β-adrenoceptor function of human peripheral blood mononuclear cells and guinea pig trachea. J. Allergy Clin. Immunol..

[109] Seyedi S., Andalib A., Rezaei A., Hosseini S.M., Mohebbi S.R., Zali M.R., Vafai M., Behboo R., Tabatabaei S.A., Shahabi S. (2012). The effects of isoproterenol and propranolol on cytokine profile secretion by cultured tumor-infiltrating lymphocytes derived from colorectal cancer patients. Cell J..

[110] Mohede I.C., van Ark I., Brons F.M., van Oosterhout A.J., Nijkamp F.P. (1996). Salmeterol inhibits interferon-gamma and interleukin-4 production by human peripheral blood mononuclear cells. Int. J. Immunopharmacol..

[111] Guha M., Mackman N. (2001). LPS induction of gene expression in human monocytes. Cell Signal..

[112] Osawa Y, Lee H.T., Hirshman C.A., Xu D., Emala C.W. (2006). Lipopolysaccharide-induced sensitization of adenylyl cyclase activity in murine macrophages. Am. J. Physiol. Cell Physiol..

[113] Liu Z., Jiang Y., Li Y., Wang J., Fan L., Scott M.J, Xiao G., Li S., Billiar T,R., Wilson M.A., Fan J. (2013). TLR4 Signaling augments monocyte chemotaxis by regulating G protein-coupled receptor kinase 2 translocation. J. Immunol..

[114] Loniewski K., Shi Y., Pestka J., Parameswaran N. (2008). Toll-like receptors differentially regulate GPCR kinases and arrestins in primary macrophages. Mol. Immunol..

[115] Johnson J.A., Clark R.B., Friedman J., Dixon R.A., Strader C.D. (1990). Identification of a specific domain in the β-adrenergic receptor required for phorbol ester-induced inhibition of catecholamine-stimulated adenylyl cyclase. Mol. Pharmacol..

[116] Yuan N., Friedman J., Whaley B.S., Clark R.B. (1994). cAMP-dependent protein kinase and protein kinase C consensus site mutations of the β-adrenergic receptor. Effect on desensitization and stimulation of adenylyl cyclase. J. Biol. Chem..

[117] Carmena M.J., García-Paramio P., Solano R.M., Prieto J.C. (1995). Protein kinase C regulation of the adenylyl cyclase system in rat prostatic epithelium. Prostate.

[118] Deiss K., Kisker C., Lohse M.J., Kristina Lorenz K. (2012). Raf kinase inhibitor protein (RKIP) dimer formation controls its target switch from Raf1 to G protein-coupled receptor kinase (GRK) 2. J. Biol. Chem..

[119] Lorenz K., Lohse M.J., Quitterer U. (2003). Protein kinase C switches the Raf kinase inhibitor from Raf-1 to GRK-2. Nature.

[120] De Blasi A., Parruti G., Sallese M. (1995). Regulation of G protein-coupled receptor kinase subtypes in activated T lymphocytes. Selective increase of beta-adrenergic receptor kinase 1 and 2. J. Clin. Investig..

[121] Pronin A.N., Benovic J.L. (1997). Regulation of the G protein-coupled receptor kinase GRK5 by protein kinase C. J. Biol. Chem..

[122] Li Q., Verma IM. (2002). NF-κB regulation in the immune system. Nat. Rev. Immunol..

[123] Wang Y., Ding M., Chaudhari S., Ding Y., Yuan J., Stankowska D., He S., Krishnamoorthy R., Cunningham J.T., Ma R. (2013). Nuclear factor κB mediates suppression of canonical transient receptor potential 6 expression by reactive oxygen species and protein kinase C in kidney cells. J. Biol. Chem..

[124] Lee B.H., Kim M.S., Rhew J.H., Park R.W., de Crombrugghe B., Kim I.S. (2000). Transcriptional regulation of fibronectin gene by phorbol myristate acetate in hepatoma cells: A negative role for NF-κB. J. Cell. Biochem..

[125] Islam K.N., Koch W.J. (2012). Involvement of nuclear factor κB (NF-κB) signaling pathway in regulation of cardiac G protein-coupled receptor kinase 5 (GRK5) expression. J. Biol. Chem..

[126] Tan K.S., Nackley A.G., Satterfield K., Maixner W., Diatchenko L., Flood P.M. (2007). β_2_-adrenergic receptor activation stimulates pro-inflammatory cytokine production in macrophages via PKA- and NF-κB-independent mechanisms. Cell Signal..

[127] Rajagopal S., Ahn S., Rominger D.H., Gowen-MacDonald W., Lam C.M., Dewire S.M., Violin J.D., Lefkowitz R.J. (2011). Quantifying ligand bias at seven-transmembrane receptors. Mol. Pharmacol..

[128] Drake M.T., Violin J.D., Whalen E.J., Wisler J.W., Shenoy S.K., Lefkowitz R.J. (2008). β-Arrestin-biased agonism at the β_2_-adrenergic receptor. J. Biol. Chem..

[129] Kin N.W., Sanders V.M. (2006). It takes nerve to tell T and B cells what to do. J. Leukoc. Biol..

[130] Swanson M.A., Lee W.T., Sanders V.M. (2001). IFN-γ production by Th1 cells generated from naive CD4+ T cells exposed to norepinephrine. J. Immunol..

[131] Badou A., Savignac M., Moreau M., Leclerc C., Foucras G., Cassar G., Paulet P., Lagrange D., Druet P., Guéry J.C. (2001). Weak TCR stimulation induces a calcium signal that triggers IL-4 synthesis, stronger TCR stimulation induces MAP kinases that control IFN-gamma production. Eur. J. Immunol..

[132] Zhang S., Kaplan M.H. (2000). The p38 mitogen-activated protein kinase is required for IL-12-induced IFN-γ expression. J. Immunol..

[133] Lubahn C.L., Lorton D., Schaller J.A., Sweeney S.J., Bellinger D.L. (2014). Targeting α- and β-adrenergic receptors differentially shifts Th1, Th2, and inflammatory cytokine profiles in immune organs to attenuate adjuvant arthritis. Front. Immunol..

[134] Vroon A, Heijnen C.J., Kavelaars A. (2006). GRKs and arrestins: Regulators of migration and inflammation. J. Leukoc. Biol..

[135] Lombardi M.S., Kavelaars A., Cobelens P.M., Schmidt R.E., Schedlowski M., Heijnen C.J. (2001). Adjuvant arthritis induces down-regulation of G protein-coupled receptor kinases in the immune system. J. Immunol..

[136] Nakaya M., Tajima M., Kosako H., Nakaya T., Hashimoto A., Watari K., Nishihara H., Ohba M., Komiya S., Tani N. (2013). GRK6 deficiency in mice causes autoimmune disease due to impaired apoptotic cell clearance. Nat. Commun..

[137] Raghuwanshi S.K., Smith N., Rivers E.J., Thomas A.J., Sutton N., Hu Y., Mukhopadhyay S, Chen X.L., Leung T., Richardson R.M. (2013). G protein-coupled receptor kinase 6 deficiency promotes angiogenesis, tumor progression, and metastasis. J. Immunol..

[138] Murga C., Mayor F. (2009). GRK6, a gatekeeper of visceral hyperalgesia. Brain Behav. Immun..

[139] Eijkelkamp N., Heijnen C.J., Carbajal A.G., Willemen H.L., Wang H., Minett M.S., Wood J.N., Schedlowski M., Dantzer R., Kelley K.W. (2012). G protein-coupled receptor kinase 6 acts as a critical regulator of cytokine-induced hyperalgesia by promoting phosphatidylinositol 3-kinase and inhibiting p38 signaling. Mol. Med..

[140] Managò F., Espinoza S., Salahpour A., Sotnikova T.D., Caron M.G., Premont R.T., Gainetdinov R.R. (2012). The role of GRK6 in animal models of Parkinson’s disease and L-DOPA treatment. Sci. Rep..

